# The Synthesis and Assembly of a Truncated Cyanophage Genome and Its Expression in a Heterogenous Host

**DOI:** 10.3390/life12081234

**Published:** 2022-08-15

**Authors:** Shujing Liu, Jia Feng, Tao Sun, Bonan Xu, Jiabao Zhang, Guorui Li, Jianting Zhou, Jianlan Jiang

**Affiliations:** 1 School of Chemical Engineering & Technology, Tianjin University, Tianjin 300072, China; 2 Key Laboratory of Systems Bioengineering (Ministry of Education), Tianjin University, Tianjin 300072, China; 3 Center for Biosafety Research and Strategy, Tianjin University, Tianjin 300072, China; 4 Frontier Science Center for Synthetic Biology (Ministry of Education), Tianjin University, Tianjin 300072, China

**Keywords:** artificial cyanophage genome, cyanophage A-4L, heterologous expression, non-host cyanobacteria, salt stress, transcriptomic analysis

## Abstract

Cyanophages play an important role in regulating the dynamics of cyanobacteria communities in the hydrosphere, representing a promising biological control strategy for cyanobacterial blooms. Nevertheless, most cyanophages are host-specific, making it difficult to control blooming cyanobacteria via single or multiple cyanophages. In order to address the issue, we explore the interaction between cyanophages and their heterologous hosts, with the aim of revealing the principles of designing and constructing an artificial cyanophage genome towards multiple cyanobacterial hosts. In the present study, we use synthetic biological approaches to assess the impact of introducing a fragment of cyanophage genome into a heterologous cyanobacterium under a variety of environmental conditions. Based on a natural cyanophage A-4L genome (41,750 bp), a truncated cyanophage genome Syn-A-4-8 is synthesized and assembled in *Saccharomyces cerevisiae*. We found that a 351–15,930 bp area of the A-4L genome has a fragment that is lethal to *Escherichia coli* during the process of attempting to assemble the full-length A-4L genome. Syn-A-4-8 was successfully introduced into *E. coli* and then transferred into the model cyanobacterium *Synechococcus elongatus* PCC 7942 (Syn7942) via conjugation. Although no significant phenotypes of Syn7942 carrying Syn-A-4-8 (LS-02) could be observed under normal conditions, its growth exhibited a prolonged lag phase compared to that of the control strain under 290-millimolar NaCl stress. Finally, the mechanisms of altered salt tolerance in LS-02 were revealed through comparative transcriptomics, and *ORF25* and *ORF26* on Syn-A-4-8 turned out to be the key genes causing the phenotype. Our research represents an important attempt in designing artificial cyanophages towards multiple hosts, and offers new future insights into the control of cyanobacterial blooms.

## 1. Introduction

Filamentous cyanobacteria are distributed all over the world, and their overbreeding results in harmful cyanobacterial blooms [1,2,3,4,5,6]. Recently, most sequenced cyanophages are viruses that infect the unicellular cyanobacteria of the *Synechococcus* and *Prochlorococcus* genera [7,8,9,10,11]. Few studies focus on the genome sequencing of cyanophages that infect freshwater filamentous cyanobacteria [12,13,14]. Additionally, in recent decades, water eutrophication and global climate warming have intensified the frequency of cyanobacterial blooms, especially in freshwater systems [6,15,16,17]. Toxin-producing cyanobacterial blooms frequently occur in many eutrophic lakes, ponds and rivers in the world [15], raising the emergency status concerning their control [6,18,19]. With a plentiful number of species and their abundance in the hydrosphere [20,21], cyanophages are a class of viruses that can infect and lyse cyanobacteria, playing an important role in regulating the dynamics of cyanobacterial communities [21,22]. Given their high efficiency, controlling cyanobacterial blooms based on cyanophages has been considered an important and promising strategy.

However, most natural cyanophages are host-specific, making it difficult to use them in large-scale applications for the control of cyanobacterial blooms due to the lack of broad-spectrum cyanophages. Most cyanophages with host specificities have different host ranges, including specialist cyanophages with a narrow host range and generalist cyanophages with a wide host range [8,23,24]. Regarding the specific infection of a tailed cyanophage (tailed phage), the specific binding of the receptor-binding protein (RPB) to the receptor on the host cell’s surface is necessary [25,26]. In addition, there are other mechanisms that contribute to explaining the infecting specificity of cyanophages. In 2019, the defense mechanisms of marine cyanobacteria *Synechococcus* and *Prochlorococcus* against T7- and T4-like cyanophages were systematically studied. The following behaviors were observed: (1) most specialist cyanophages could not attach to the surfaces of non-host cyanobacterial cells, resulting in a failed infection, and (2) most generalist cyanophages could inject their genomic DNA into non-host cyanobacteria cells, but non-host cyanobacteria hindered virus production through various intracellular interactions [24]. This may be the factor that is relevant to the infection specificity of cyanophages. However, due to the genomic DNA of cyanophages that cannot be injected into phylogenetically distant non-host cyanobacteria naturally, there are still few studies on the intracellular resistance of non-host cyanobacteria to cyanophages, as well as studies on the effects of cyanophages on non-host cyanobacteria.

With the development of synthetic biology, the artificial synthesis of various genomes has been realized, such as the chemical synthesis of the *Mycoplasma mycoides* genome [27,28], recoding of the *Escherichia coli* genome [29], artificial synthesis of *Saccharomyces cerevisiae* chromosomes [30,31,32] and a data-carrying chromosome [33,34]. Using rational design, DNA synthesis and assembly as well as non-natural functions such as degenerate codons and truncated genomes have been introduced into modified microorganisms, providing new insights into the design and synthesis of artificial cyanophages with a broad spectrum. Although artificial synthesis of the ø X174, T7 and AP205 phages has been achieved [35,36,37,38], the synthesis of the full-length cyanophage genome has not yet been reported.

**Figure 1:**
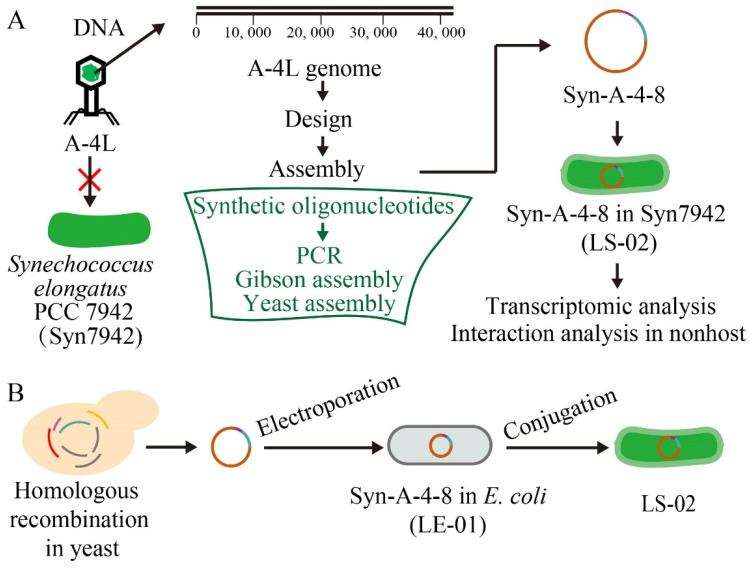
Schematic of the present study. (**A**) The general idea of this study. Syn-A-4-8 truncated cyanophage genome. (**B**) Schematic diagram of plasmid construction and transformation in this study. *Synechococcus elongatus* PCC 7942 (Syn7942).

In order to solve the limitation of the host specificity of natural cyanophages in their application to control cyanobacterial blooms, we aim to explore the principles of designing and constructing an artificial cyanophage in relation to multiple cyanobacterial hosts. The freshwater cyanophage A-4L belongs to the Podoviridae family in the Caudovirales order, and only infects some specific strains of genera of *Nostoc* sp. [39]. The A-4L genome is a linear double-stranded DNA with a length of 41,750 bp, containing 810 bp of direct terminal repeats and encoding 38 open reading frames (ORFs) from *ORF1* to *ORF38* [40]. Model cyanobacterium *Synechococcus elongatus* PCC 7942 (hereafter Syn7942) is a single-cell model cyanobacterium with a genome of approximately 2.7 Mb [41]. Syn7942 has a well-established genetic operation system, and the target plasmids can be transferred into *Synechococcus* using natural transformation or conjugation [42,43,44]. In this study, we synthesize and assemble a truncated cyanophage genome Syn-A-4-8 in order to investigate the effect of an artificial cyanophage genome on non-host cyanobacteria by transferring it into Syn7942 via conjugation (Figure 1). Although no significant phenotypes of Syn7942 carrying Syn-A-4-8 (LS-02) could be observed in normal conditions, its growth exhibited a prolonged lag phase compared to that of the control strain under 290-millimolar NaCl stress. The related mechanisms in LS-02 were revealed using comparative transcriptome analysis, and the key genes on Syn-A-4-8 causing the phenotype were also revealed. Our study lays the foundation for a further construction of a broad-spectrum artificial cyanophage, and provides new future insights into the control of cyanobacterial blooms.

## 2. Materials and Methods

### 2.1. Strains, Plasmids and Culture Conditions

All strains and plasmids used in this study are presented in Table S1. Competent cells of *E. coli* DH10B (NEB 10-beta electrocompetent cells) used in this study were purchased from Biomed, China. All *E. coli* strains were cultured in LB medium at 37 °C with antibiotics when needed (50 μg/mL kanamycin). Wild-type *S. cerevisiae* BY4741 was grown in YPD medium at 30 °C. Yeast transformants were selected and cultured on synthetic complete medium without leucine (SC-Leu) or SC-His medium.

All Syn7942 strains constructed in this study and the plasmids used are presented in Table 1. All *Synechococcus* strains were cultured at 37 °C with a light density of 100 μmol/(m^2^·s) under a normal culture condition [45]. Wild-type Syn7942 was grown in BG11 medium, and constructed *Synechococcus* strains were cultured in BG11 medium supplemented with 25 μg/mL of kanamycin. Cyanobacteria live in different natural environments, such as freshwater, seawater and estuaries. Therefore, some stress culture conditions were chosen to simulate various adverse natural environments and observe the growth of Syn7942 strains, including different light intensities, different temperatures, nitrogen deficiency and salt stress (Table S2). The fresh seed liquid was inoculated into 20 mL of fresh liquid medium that was supplemented with 25 μg/mL of kanamycin. The optical density (OD) at 750 nm and the full absorption spectrum of liquid cultures were measured by using a Varioskan LUX multifunctional microplate reader (Thermo Fisher Scientific, Waltham, MA, USA). The initial OD_750_ of the liquid culture was 0.04, and it was measured every 12 h. The full absorption spectrum of the strain cells was measured at 72 h. Each strain was repeated by three biological parallel samples. For nitrogen depletion and salt treatment, BG-11 medium without sodium nitrate (BG-N^−^) and BG11 medium supplemented with salt (190 mM NaCl or 290 mM NaCl) were applied to analyze the growth curves of *Synechococcus* strains, respectively.

### 2.2. Construction of Truncated Cyanophage Genome and Plasmid

The truncated cyanophage genome Syn-A-4-8 (34,770 bp) was designed based on the cyanophage A-4L genome sequence (GenBank: KF356198, 41,750 bp). The A-4L gene sequence was divided into eight DNA fragments with lengths of approximately 5 kb (A1–A8). The five large DNA fragments (A4–A8) were, respectively, assembled in *E. coli* using pUC57 plasmid as a vector. Additionally, *Not* I restriction sites (5′-GCGGCCGC-3′) were added at both ends of the large DNA fragment in order to connect the pUC57 vector. The *E. coli*-cyanobacteria shuttle plasmid pJA2 [46] was used as the backbone vector for Syn-A-4-8. Additionally, a yeast replication element, *CEN6/ARS4*, and a *His3* marker from pLS0 plasmid were added to enable Syn-A-4-8 to be replicated and screened in *S. cerevisiae*. The pLS0 plasmid is a retrofitted BAC-YAC shuttle plasmid in our lab. Its plasmid map is shown in Figure S1, and the specific DNA sequences of *CEN6/ARS4* and *His3* are shown in Table S3. The five large DNA fragments (A4–A8) from enzyme digestion, linearized vector pJA2, yeast replication element *CEN6/ARS4* and *His3* marker were co-transformed into *S. cerevisiae*, realizing the one-step assembly of the truncated cyanophage genome Syn-A-4-8 by the LiAc/SS carrier DNA/PEG method [47]. Syn-A-4-8 carries 14 ORFs from *ORF25* to *ORF38* of the A-4L genome. The obtained yeast transformants were verified using colony PCR and DNA sequencing. The specific primers involved are shown in Table S4.

Similarly, four recombinant plasmids using the same vector backbone as Syn-A-4-8 were constructed in *S. cerevisiae*. More specifically, the recombinant plasmid pSJ03 contains the *ORF25*, *ORF26*, *ORF27* and *ORF38* of A-4L; pSJ04 contains the *ORF25* and *ORF26* of A-4L; pSJ05 contains the *ORF25* of A-4L; and pSJ06 contains the *ORF26* of A-4L. The recombinant plasmids were verified using PCR sequencing and the specific primers involved are shown in Table S4.

### 2.3. Transformation of Syn7942

The best way to transfer Syn-A-4-8 into Syn7942 is by conjugation rather than by natural transformation, due to the size of Syn-A-4-8 (34,770 bp). *Synechococcus elongatus* UTEX 2973 (hereafter Syn2973) is a close relative of Syn7942 [48]. The target genome Syn-A-4-8 was transferred into Syn7942 by conjugation, according to the transformation method used in Syn2973 [43,44]. The truncated cyanophage genome Syn-A-4-8 was transferred into Syn7942 from *E. coli* under the combined action of pRL443 (conjugative plasmid) and pRL623 (helper plasmid).

The target plasmid was electroporated into NEB 10-beta electrocompetent cells (Biomed, Beijing, China) as the donor strain. Briefly, 2 mL of mid-log-phase *E. coli* containing the target plasmid and 2 mL of mid-log-phase *E. coli* HB101 containing pRL443 and pRL623 were washed twice with antibiotic-free LB medium and then separately resuspended in 100 μL of LB medium that was fully mixed and incubated at 37 °C for 30 min. A total of 1 mL of mid-log-phase Syn7942 cells were centrifuged and resuspended in 0.2 mL of BG11 medium, mixed with the above suspension and incubated at 37 °C for 30 min. The mixed cells were plated onto 0.45-micrometer pore-size cellulose nitrate membranes on BG11 + 5% LB (*v*/*v*) agar plates and incubated at 37 °C for 24 h in 100 μmol/(m^2^·s) light. Then, the membrane was transferred to BG11 agar plates with 25 μg/mL of kanamycin. After incubation at 37 °C for 5–7 days, cyanobacterial transformants were observed. These single transformants were then streaked onto selective medium plates again to select stable transformants. Cyanobacterial transformants were verified by colony PCR and Sanger sequencing. The specific primers involved are shown in Table S4.

### 2.4. Transcriptomic Analysis

In order to explore the heterologous expression of Syn-A-4-8 in Syn7942 and its effect on the heterologous host, transcriptomic analysis was conducted on constructed Syn7942 strains cultured for 72 h under normal conditions and 290-millimolar NaCl stress. Transcriptomic analysis was performed using the RNA-sequencing method (RNA-seq) in GENEWIZ (Suzhou, China). Three biological replicates were performed for each sample. After the quality assessment of raw reads obtained by transcriptome sequencing, low-quality reads were pre-processed to obtain clean reads. The filtered sequenced clean data were compared with the reference genome (GenBank: CP000100.1). The gene expression level was calculated using the FPKM (fragments per kilobases per million reads) method using Htseq software (V 0.6.1) [49,50]. Based on the data of the experimental and the control groups, DESeq2 (V1.6.3) of the Bioconductor software was used for differential gene expression analysis [51]. Differentially expressed genes (DEGs) were screened according to a fold change > 1.5 and *p*-value < 0.05.

### 2.5. Quantitative Real-Time PCR (qRT-PCR)

After being cultured in liquid medium for 72 h, the cell culture (volume*OD_750nm_ = 5) was collected by centrifugation and then immediately frozen with liquid nitrogen. Then, total RNA was extracted from the cells using the Direct-zol^TM^RNA MiniPrep kit (Zymo, Irvine, CA, USA) according to the manufacturer’s instructions. Total RNA was reverse transcribed into cDNA using the HiScript^®^ II Q RT SuperMix for qPCR (+gDNA wiper; Vazyme, Nanjing, China) according to the manufacturer’s instructions. cDNA was diluted to 1/100 for qRT-PCR templates. The qRT-PCR reaction system was prepared with the ChamQ Universal SYBR qPCR Master Mix (Vazyme, Nanjing, China) according to the manufacturer’s instructions. Additionally, qRT-PCR was performed using the StepOne Real-time PCR system (Applied Biosystems, Foster City, CA, USA). According to a reported method [52], qRT-PCR was used to evaluate the level of heterologous expression of the truncated cyanophage genome Syn-A-4-8 in Syn7942. The primers used for qRT-PCR are presented in Table S4. The *rnpB* gene encoding RNase P subunit B was used as an internal reference gene. Three technical replicates were performed for each sample. The qRT-PCR data were analyzed by using StepOne Software (Applied Biosystems, Foster City, CA, USA) and the 2^−ΔΔCt^ method [53].

The reliability of the RNA-seq expression data was validated by the qRT-PCR of 18 selected genes using the same RNA extractions for RNA-seq. The primers used for verification are presented in Table S4. Similarly, three technical replicates were performed for each sample. The correlations between RNA-seq and qRT-PCR were assessed by means of scatter graphs as well as the Pearson correlation coefficient in Excel. If the correlation coefficient was greater than 0.8, the RNA-seq expression data were considered reliable.

## 3. Results

### 3.1. Synthesis and Assembly of a Truncated Cyanophage Genome Syn-A-4-8

**Figure 2:**
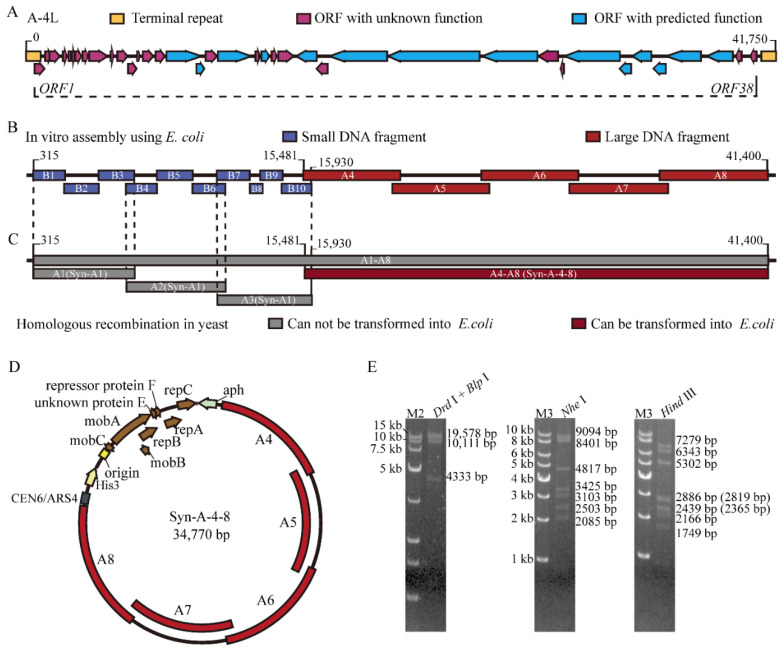
Synthesis and assembly of the truncated cyanophage genome Syn-A-4-8. (**A**) The natural cyanophage A-4L contains genes *ORF*1–*ORF*38. (**B**) Results of DNA fragment assembly using *E. coli*. The DNA fragments (B1–B10) cannot be assembled into larger DNA fragments in *E. coli*. (**C**) Recombinant plasmids and artificial genomes assembled by homologous recombination in yeast. Syn-A1, pRS415 harboring the A1 cassette; Syn-A2, pRS415 harboring the A2 cassette; Syn-A3, pRS415 harboring the A3 cassette; Syn-A-4-8, the truncated cyanophage genome. (**D**) Map of artificial genome Syn-A-4-8. Syn-A-4-8 contains 14 ORFs from ORF25 to ORF38 in the A-4L genome. (**E**) The gel electrophoresis results of restriction enzyme digestion for Syn-A-4-8. M2: *Trans*15K DNA marker. M3: 1 kb DNA ladder. *Drd* I + *Blp*: Syn-A-4-8 was digested with the restriction endonucleases *Drd* I and *Blp* I and then divided into four fragments (19,578 bp, 10,111 bp, 4333 bp, 748 bp). *Nhe*: Syn-A-4-8 was digested with the restriction endonuclease *Nhe* I and then divided into ten fragments (9094 bp, 8401 bp, 4817 bp, 3425 bp, 3103 bp, 2503 bp, 2085 bp, 841 bp, 385 bp, 116 bp). *Hind* III: Syn-A-4-8 was digested with the restriction endonuclease *Hind* III and then divided into twelve fragments (7279 bp, 6343 bp, 5302 bp, 2886 bp, 2819 bp, 2439 bp, 2365 bp, 2166 bp, 1749 bp, 915 bp, 321 bp, 187 bp).

In order to synthesize the truncated cyanophage genome Syn-A-4-8 based on the natural cyanophage A-4L (GenBank: KF356198, 41,750 bp, containing genes including *ORF1*–*ORF38*, infecting filamentous *Nostoc* sp. PCC 7120) (Figure 2A), firstly, eight DNA fragments (A1–A8) of the A-4L genome were tentatively assembled and cloned in *E. coli* from synthetic oligonucleotides. Surprisingly, the results show that DNA fragments A1, A2 and A3 could not be successfully cloned in *E. coli*, whereas they could survive separately in *E. coli* with 10 small DNA fragments (B1–B10) with lengths of 1–2 kb (Figure 2B). The DNA fragments A1, A2 and A3 were successfully assembled by homologous recombination in *S. cerevisiae* using a shuttle vector pRS415 containing a colE1 replication origin and a yeast replication element *CEN/ARS* with a *Leu2* marker [54], leading to the construction of Syn-A1, Syn-A2 and Syn-A3 (Figure 2C). Nevertheless, Syn-A1, Syn-A2 and Syn-A3 could still not be introduced into *E. coli* (Table S5), suggesting the existence of toxic genes.

Given the potential toxicity of fragments from A1 to A3 (Figure 2C), the truncated cyanophage genome Syn-A-4-8 containing A4–A8 was successfully assembled in *S. cerevisiae* and validated (Figure 2D,E). In this case, the *E. coli*-cyanobacteria shuttle plasmid pJA2 [46] with a yeast replication element, *CEN6/ARS4*, and a *His3* marker from the pLS0 plasmid, were used as the backbone. As illustrated in Table S6, Sanger sequencing shows that Syn-A-4-8 contains five base mutations. Unlike the results of Syn-A1, Syn-A2 and Syn-A3, Syn-A-4-8 could be transferred into *E. coli* via electroporation (Table S5), suggesting the existence of toxic genes in fragments from A1 to A3.

### 3.2. Heterogeneous Expression and Investigation of Syn-A-4-8 in Syn7942

Although many cyanobacterial species are naturally transformable [55], the introduction of large plasmids (>20 kb) mainly depends on the conjugation mediated by *E. coli* [56]. We focused on the heterogeneous expression and investigation of Syn-A-4-8 in non-host cyanobacterium Syn7942. As expected, Syn-A-4-8 was successfully transferred into Syn7942 by *E. coli*-mediated conjugation, leading to the LS-02 test strain (Table 1). Meanwhile, the LS-01 control strain, which carried empty plasmid pJA2 in Syn7942, was also constructed (Table 1). The cyanobacteria transformants were verified by colony PCR screening (Figure S2).

**Figure 3:**
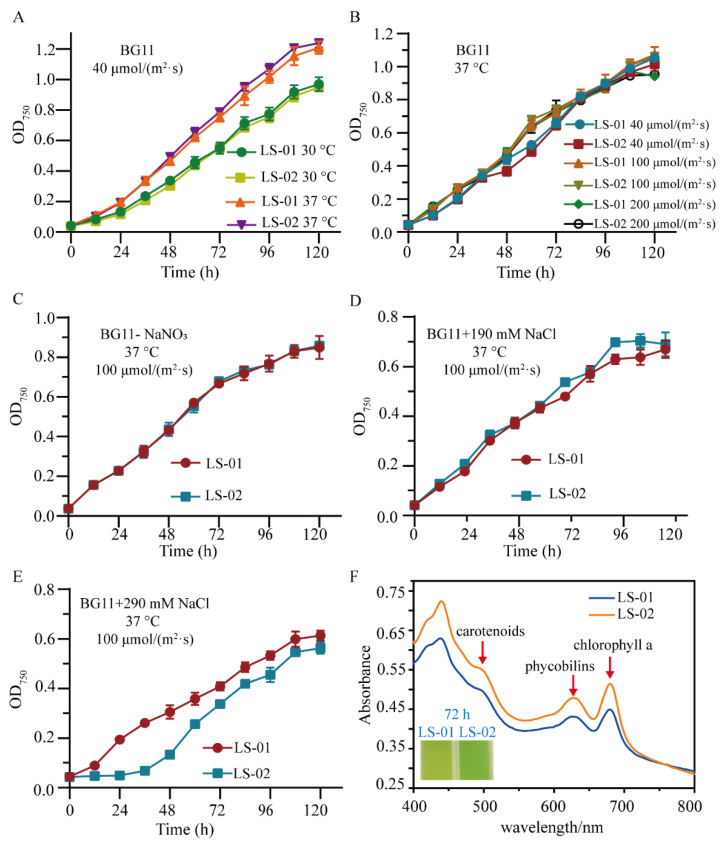
The transfer of Syn-A-4-8 had a minor effect on Syn7942 under various culture conditions. The error bars represent the standard deviations of the three biological replicates for each sample. (**A**) The growth curves of LS-01 and LS-02 under the same light intensities at different culture temperatures. (**B**) The growth curves of LS-01 and LS-02 at 37 °C under different light densities. (**C**) The growth curves of LS-01 and LS-02 in BG11 medium without sodium nitrate. (**D**) The growth curves of LS-01 and LS-02 in BG11 liquid medium supplemented with salt (190 mM NaCl). (**E**) The growth curves of LS-01 and LS-02 in BG11 liquid medium supplemented with salt (290 mM NaCl). (**F**) Full absorption spectrum of strain cells in BG11 liquid medium supplemented with salt (290 mM NaCl).

The LS-02 and LS-01 strains were cultured under a variety of culture conditions to investigate the effect of Syn-A-4-8 on Syn7942 (Table S2). Under different light intensities, different temperatures, the growth curve of the LS-02 test strain was similar to that of the LS-01 control strain (Figure 3A,B). As Syn7942 has no nitrogen fixation capacity, the growth of LS-01 and LS-02 under nitrogen-deficiency conditions was both slightly weaker than that under the normal culture condition, but the growth of LS-02 was consistent with that of LS-01 (Figure 3C). Additionally, absorption spectra also showed that the cytochrome content between LS-02 and LS-01 presented a minor difference under nitrogen-deficiency conditions (Figure S3A). Although the absorption spectra showed that the cytochrome content between LS-02 and LS-01 presented a slight difference under 190-millimolar NaCl stress (Figure S3B), the growth of LS-02 was similar to that of LS-01 (Figure 3D). Therefore, we tried a higher concentration of salt stress culture.

Interestingly, the growth of LS-02 exhibited a prolonged lag phase compared to that of LS-01 under 290-millimolar NaCl stress (Figure 3E). The absorption spectrum revealed that the quantities of chlorophyll a (680 nm) [57], carotenoid (approximately 505 nm) and phycocyanin (625 nm) in the test-strain LS-02 cells were higher than those in the control-strain LS-01 cells under 290-millimolar NaCl stress (Figure 3F). All the results suggest that the salt tolerance of LS-02 was altered due to the expression of Syn-A-4-8.

### 3.3. Transcriptional Analysis Revealing the Effect of Syn-A-4-8 on Syn7942

The growth of LS-02 exhibited a prolonged lag phase compared to that of LS-01 under 290-millimolar NaCl stress. In order to reveal the effect of mechanisms of the prolonged lag phase in LS-02, transcriptomic analysis was performed for LS-02 and LS-01 under both normal and salt stress conditions. The reliability of the transcriptomic data was validated using qRT-PCR testing, reaching a correlation of >0.9 (Figure S4).

#### 3.3.1. Transcriptomic Comparations of LS-02 and LS-01 under Normal Conditions

**Figure 4:**
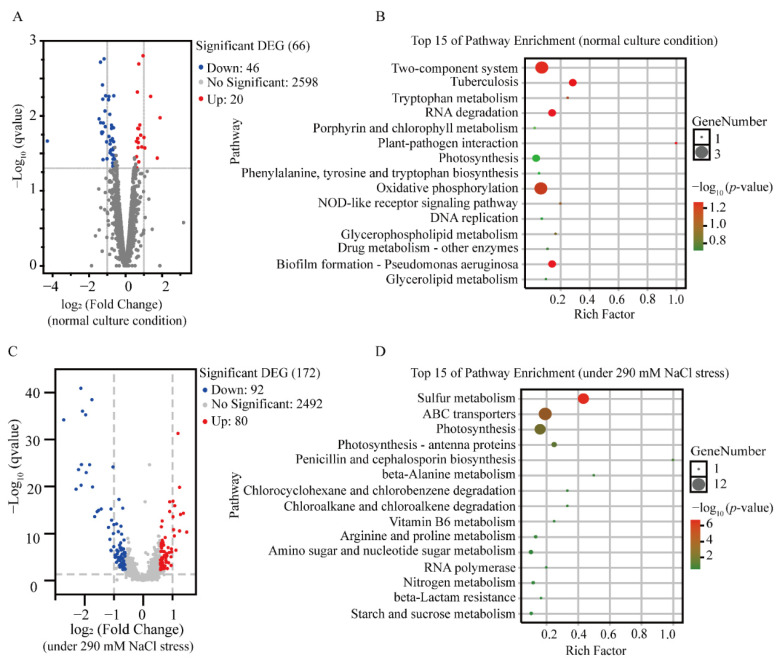
Response of Syn7942 cells to heterologous expression of Syn-A-4-8. (**A**) Volcano map of differentially expressed genes under normal culture conditions. Red dots indicate up-regulation and blue dots indicate down-regulation. The abscissa represents the logarithm of the fold change of gene expression, and the ordinate represents the statistical significance of the change in gene expression. Differentially expressed genes (DEGs) were screened based on a fold change  >  1.5 and *p*-value < 0.05. (**B**) KEGG pathway-enrichment scatter plot under normal culture conditions. The abscissa represents the Rich factor. The larger the Rich factor, the greater the enrichment degree. The ordinate represents the pathway name. The size of each dot indicates the number of DEGs in this pathway, and the color of each dot corresponds to different *p*-values. The smaller the *p*-value, the more significant the enrichment. (**C**) Volcano map of differentially expressed genes under 290-millimolar NaCl stress. (**D**) KEGG pathway-enrichment scatter plot under 290-millimolar NaCl stress.

Although the growth of LS-02 was consistent with that of LS-01, the heterologous expression of Syn-A-4-8 resulted in differentially expressed genes (DEGs) under normal culture conditions. As a result, a total of 2664 expressed genes of Syn7942 were identified under the normal culture condition, of which 66 DEGs, including 46 down-regulated and 20 up-regulated genes, were screened in LS-02 compared to those in LS-01 using a 1.5-fold change as a cutoff (Figure 4A). According to pathway enrichment, the enriched DEGs were mainly involved in oxidative phosphorylation, two-component systems, heat shock proteins (HSPs), photosynthesis and transporters (Figure 4B, Table S7).

Three down-regulated genes were involved in oxidative phosphorylation (Table S7), including *ctaA* encoding heme-A synthase (HAS) and two genes (*coxA* and *coxC*) encoding subunits I (COI) and III (COIII) of cytochrome-c oxidase. Cyanobacteria are oxygenic photosynthetic organisms, and the photosynthetic and respiratory electron transport chains are located in the thylakoid membrane (TM), forming a metabolically active membrane network and maintaining redox balance [58,59]. Terminal oxidases in the respiratory chain, the key enzymes for cyanobacterial respiration, accept electrons and reduce molecular oxygen to water, as well as generate a proton motive force used for ATP synthesis [60,61]. Cytochrome-c oxidase is a heme-copper oxidase, and its important cofactor, heme A, is synthesized by HAS [62,63]. Additionally, in *Synechocystis* sp. PCC 6803, terminal oxidases of the respiratory chain can alleviate the photo-oxidative damage of photosystem I under repetitive short-pulse illumination [64]. As previously reported, the expression of ubiquinone oxidoreductase in the respiratory chain of host cyanobacteria *Nostoc* sp. FACHB-596 decreased after being infected with cyanophage YongM at 8 h, limiting proton transport during host oxidative phosphorylation [65]. Thus, the down-regulation of the three genes in our study may adversely affect electron transfer in the respiratory chain of cells, resulting in the weakening of cyanobacterial respiration.

The two-component system involved three down-regulated genes encoding HAS (Table S7), CheW protein and CheY-like protein. In *Bacillus*, cells sense the hypoxia signal through the ResDE two-component regulatory system and improve the biosynthesis of terminal oxidases; terminal oxidases interact with histidine kinase *kinB* to activate the formation of biofilm [66]. The cofactor heme A of terminal oxidases is synthesized by HAS. CheW and CheY-like proteins belong to the bacterial chemotactic system, an improved two-component system that responds to environmental stimuli by regulating gene expression and moving cells towards a favorable environment [67,68]. In *E. coli*, the chemotaxis signal transduction protein CheW connects methyl-accepting chemotaxis proteins (MCPs) and histidine kinase CheA to form a stable core signal complex, which is the core component of the chemotaxis system [67,69]. After the self-phosphorylation of CheA, the phosphorylated group is transferred to the reactivity regulatory protein CheY, which controls its affinity for movement through its phosphorylation state [70]. In *Synechocystis* sp. PCC 6803, there are genes (*pilG*, *pilH*, *pilI* and *pilJ*) whose products are homologous to PatA (CheY-like protein), CheY, CheW and MCPs, to participate in its phototactic motility in order to better adapt to environmental stimuli [71,72]. The down-regulation of these three genes may adversely affect the ability of cells to respond to environmental stimuli.

Five down-regulated genes were involved in HSPs (Table S7), including genes encoding IbpA (HSP20), DnaK (HSP70), HtpG (HSP90) and a DnaK/DnaJ chaperone ClpA. DnaK-DnaJ-GrpE and GroEL-GroES are two representative molecular chaperone systems present in *E. coli*, and some HSPs, including IbpA, IbpB, ClpB, ClpA and HtpG, use their co-chaperones to maintain cell protein homeostasis [73]. DnaK-DnaJ-GrpE, the HSP70 chaperone system, including DnaK (HSP70), co-chaperone DnaJ (HSP40) and GrpE (nucleotide exchange factor), play a role in maintaining protein homeostasis under physiological and stress conditions in *E. coli* [74]. The small heat shock proteins, IbpA and IbpB, bind to denatured proteins to form a complex that promotes the refolding of denatured proteins in the copolymer via DnaK/DnaJ and ClpB [75]. The high-temperature protein G (HtpG, HSP90) has been shown to be involved in stabilizing proteins required for the assembly of phycobilisomes in cyanobacteria [76]. Cyanobacteria contain multiple GroELs that jointly maintain the protein homeostasis of cells, including GroEL1, which forms an operon with the co-chaperone GroES gene and monocistronic GroEL2 [77].

Two genes involved in photosynthesis were up-regulated (Table S7), namely the *Syn7942_0407*-encoding photosystem I reaction center subunit X (PsaK) and *petG*-encoding cytochrome b6-f complex subunit 5 (PetG). PsaK, a small subunit of photosystem I complexes, contributes to photosystem I electron transport [78]. The cytochrome b6-f complex is a photosynthetic electron transport complex in thylakoid membranes which participates in electron transport from photosystem II to I and in the respiratory chain [58]. After cyanophage P-TIM68-carrying photosynthesis-related genes infect cyanobacteria MIT9515, the expressions of these cyanophage genes maintain or control the photosynthesis of host cells [79]. The up-regulation of the photosynthesis-related genes may be related to the transferred partial cyanophage genome Syn-A-4-8.

In addition, two genes encoding Na^+^/H^+^ antiporter subunits were down-regulated (Table S7). Under salt stress, the Na^+^/H^+^ antiporter actively excretes excess Na^+^ in cells and improves their salt tolerance [80,81].

#### 3.3.2. Transcriptomic Response of LS-02 to Salt Stress

Under 290-millimolar NaCl stress, a total of 2664 expressed genes of Syn7942 were also detected, of which 172 DEGs (92 down-regulated and 80 up-regulated genes) were identified in LS-02 compared to those in LS-01 using a 1.5-fold change as a cutoff value, accounting for 6.4% of the total expressed genes (Figure 4C). The DEGs were involved in various pathways, such as the sulfur metabolism of energy metabolism, ABC transporters, photosynthesis antenna proteins and photosynthesis (Figure 4D). In order to explore the growth difference caused by the heterologous expression of Syn-A-4-8 in Syn7942, the DEGs were further classified into seven groups (Table S8).

**Figure 5:**
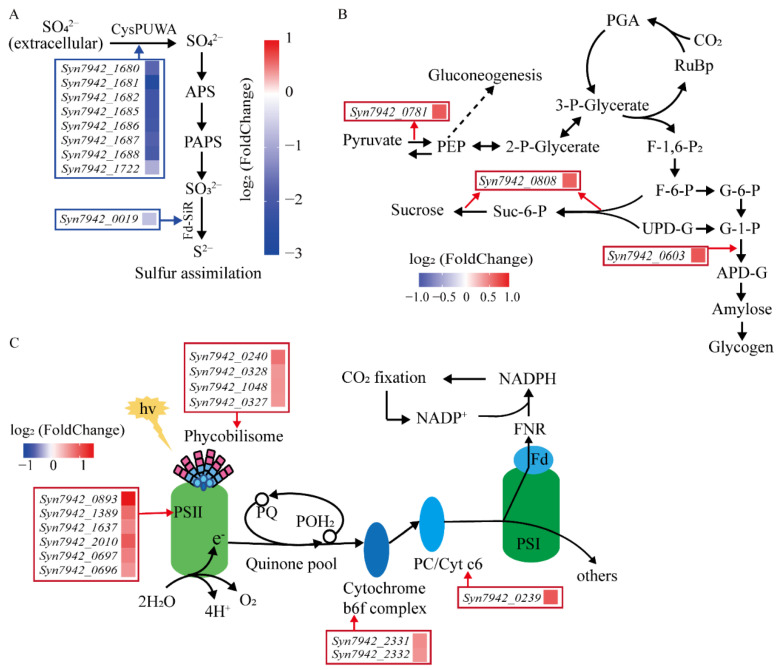
Pathways involved in DEGs. (**A**) DEGs involved in sulfur metabolism. APS: 5′-adenylylsulfate. PAPS: 3′-phosphoadenosine-5′-phosphosulfate. Fd-SiR: sulfite reductase (ferredoxin). (**B**) DEGs involved in carbohydrate metabolism. RuBP: ribulose-1,5-bisphosphate; PGA: 3-phosphoglycerate; PEP: phosphoenolpyruvate; 3-P-glycerate: 3-phospho-D-glycerate; 2-P-glycerate: 2-phospho-D-glycerate; F-1,6-P_2_: beta-D-fructose 1,6-bisphosphate; F-6-P: fructose-6-phosphate; G-6-P: glucose-6-phosphate; G-1-P: glucose-1-phosphate; ADP/UDP-G: ADP/UDP-glucose; Suc-6-P: sucrose-6-phosphate. (**C**) DEGs involved in photosynthesis antenna proteins and photosynthesis. PSI: photosystem I; PSII: photosystem II; PQ: plastoquinone; POH_2_: plastoquinol-1; PC: plastocyanin; Fd: ferredoxin; FNR: ferredoxin-NADP^+^ reductase.

Ten down-regulated genes (Group 1) were involved in sulfur metabolism, of which 8 genes encoded ATP-binding cassette (ABC) transporters of the sulfate/thiosulfate transport system and *Syn7942_0019* encoded sulfite reductase (ferredoxin) (Fd-SiR) (Table S8, Figure 5A). Sulfur, an essential element for microorganisms, enters cells in the form of inorganic sulfur through ABC transporters [82]. The redox state of cells is maintained by increasing sulfur metabolism and the biosynthesis of sulfur-containing compounds to improve salt tolerance [83]. In cyanobacteria, sulfite (S^4+^) is reduced to sulfide (S^2−^) catalyzed by Fd-SiR through the sulfate assimilation pathway for the biosynthesis of sulfur-containing amino acids [84]. The expression levels of 10 genes in group 1 related to sulfur metabolism were down-regulated, which may have affected the sulfur metabolism and biosynthesis of sulfur-containing compounds in LS-02.

Four up-regulated genes (Group 2) involved in carbohydrate metabolism (Table S8, Figure 5B), such as *Syn7942_0808* encoding sucrose phosphate synthase (SPS), *Syn7942_0603* encoding glucose-1-phosphate adenylyltransferase (GlgC) and *Syn7942_0781* encoding phosphoenolpyruvate synthase (PpsA). Syn7942 mainly accumulates sucrose as the osmotic regulator to regulate osmotic pressure under salt stress conditions [85]. PpsA catalyzes the conversion of pyruvate to phosphoenolpyruvate (PEP), the formation of which is the initial step of gluconeogenesis [86,87]. The SPS encoded by *Syn7942_0808* contains SPS and sucrose phosphate phosphatase (SPP) domains, and has bifunctional activity, which can catalyze fructose-6-phosphate and UDP-glucose to synthesize sucrose [88]. GlgC is an important regulator of bacterial glycogen biosynthesis [89,90]. In Syn7942, glycogen can be used as a carbon source for sucrose synthesis, and the *Syn7942_0603* and *Syn7942_0808* genes were both overexpressed simultaneously, which could improve the level of sucrose synthesis [91]. The up-regulation of the above-mentioned genes makes carbon metabolism in cells tend to carbohydrate synthesis; that is, towards the accumulation of energy.

Thirteen up-regulated genes (Group 3) were related to photosynthesis antenna proteins and photosynthesis (Table S8, Figure 5C). Following infection with cyanophage S-PM2, the host transcription of cyanobacteria *Synechococcus* WH7803 was not significantly closed, and the genes involved in photosynthesis were partially up-regulated [92]. After 8 h of YongM infection, phycocyanin-related rod-linked proteins of host cyanobacteria *Nostoc* sp. FACHB-596 were up-regulated, which improved the photosynthetic efficiency of the host [65]. The up-regulation of these cyanobacteria genes may be related to the transferred partial cyanophage genome Syn-A-4-8.

According to the results of the transcriptomic analysis, both DEGs under normal and salt stress conditions are involved in oxidative phosphorylation, photosynthesis and the Na^+^/H^+^ antiporter (Tables S7 and S8). These results match those observed in previous studies [65,79,92]; the infection of host cyanobacteria by the cyanophage would limit the oxidative phosphorylation of cyanobacteria and maintain or enhance the photosynthetic efficiency of the host.

### 3.4. Identification of the Genes in Syn-A-4-8 Causing the Prolonged Lag Phase of Syn7942

**Figure 6:**
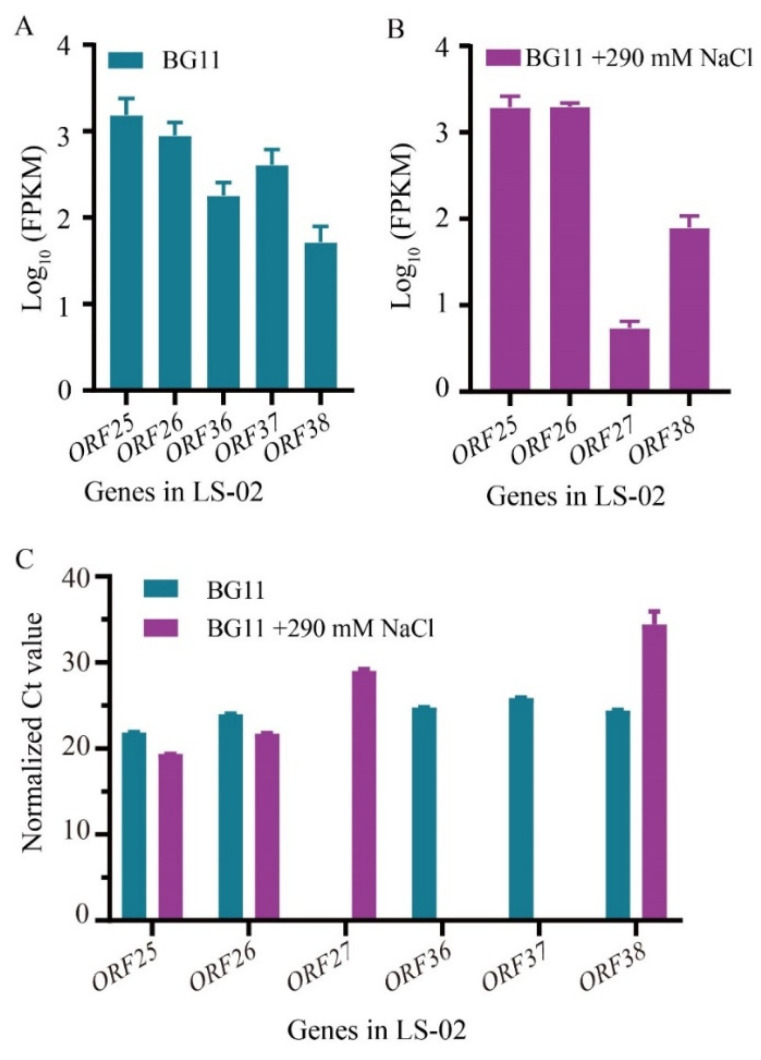
Identification of the cyanophage genes with transcripts in non-host Syn7942. The error bars represent standard deviations of the three biological replicates for each sample. (**A**) FPKM values (fragments per kilo bases per million reads) of expressed genes carried by Syn-A-4-8 in LS-02 cells according to the transcriptomics under normal culture conditions. (**B**) FPKM values of expressed genes in LS-02 cells according to the transcriptomics under 290-millimolar NaCl stress conditions. (**C**) qRT-PCR validations of the expressed genes in LS-02 under normal and salt stress. Normalized Ct value: the Ct value of each gene was normalized by that of the housekeeping gene *rnpB*.

Finally, we tried to identify the specific genes in Syn-A-4-8 that are related to the phenotype in LS-02. Although Syn-A-4-8 contained 14 ORFs (from *ORF25* to *ORF38*) originally annotated in A-4L, only some of their transcripts were detected according to the results obtained from the transcriptomic data. More specifically, the transcripts of five ORFs, including *ORF25* (hypothetical protein), *ORF26* (tail protein), *ORF36* (terminase), *ORF37* (hypothetical protein) and *ORF38* (hypothetical protein), were detected under normal conditions, while *ORF25*, *ORF26*, *ORF27* (tail protein) and *ORF38* were found under salt stress conditions (Figure 6A,B). The transcription levels of *ORF25* and *ORF26* were higher than those of *ORF27*, *ORF36*, *ORF37* and *ORF38*. The relative expressions of the ORFs in Syn-A-4-8 obtained by qRT–PCR analyses also proved the above-mentioned results (Figure 6C). Syn-A-4-8 exists in Syn7942 cells, but most of the cyanophage genes have no transcripts.

**Figure 7:**
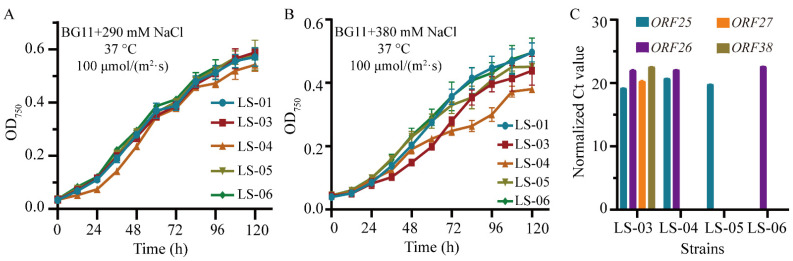
Cyanobacterial growth differences may be caused by the co-effect of *ORF25* and *ORF26*. The error bars represent standard deviations of the three biological replicates for each sample. (**A**) The growth curves of LS-01, LS-03, LS-04, LS-05 and LS-06 in BG11 liquid medium supplemented with 290 mM NaCl. (**B**) The growth curves of LS-01, LS-03, LS-04, LS-05 and LS-06 in BG11 liquid medium supplemented with 380 mM NaCl. (**C**) QRT-PCR validations of the expressed genes in LS-03, LS-04, LS-05 and LS-06. Normalized Ct value: the Ct value of each gene was normalized by that of the housekeeping gene *rnpB*.

Based on the results mentioned above, we focused on the four genes of Syn-A-4-8 expressed under the 290-millimolar NaCl condition. In this case, the LS-03 strain containing *ORF25*, *ORF26*, *ORF27* and *ORF38* was constructed (Table 1). Although the growth pattern of LS-03 was similar to that of the control strain, LS-01, under 290 mM NaCl (Figure 7A) a decreased level of growth was observed when increasing the salt concentration to 380 mM (Figure 7B), suggesting that the four ORFs were responsible for altered salt tolerance. Furthermore, *ORF27* and *ORF28* were omitted, given their relatively lower abundances, leading to the LS-04 strain containing *ORF25* and *ORF26* (Table 1). As expected, a phenotype similar to LS-03 was observed in LS-04 under 380 mM (Figure 7B). We then separately expressed *ORF25* and *ORF26* in LS-05 and LS-06, but no significant phenotypes could be investigated (Figure 7B). The transcription levels of specific genes in LS-03, LS-04, LS-05 and LS-06 were verified by qRT-PCR analyses (Figure 7C), re-confirming the relatively higher transcriptional levels of *ORF25* and *ORF26*. Based on the results above, we speculate that the decreased tolerance of LS-02 to salt stress may be caused by the co-effect of *ORF25* and *ORF26*.

## 4. Discussion

The design and assembly of the artificial cyanophage genome could represent a feasible strategy for obtaining cyanophages that can target multiple hosts, promoting the control of cyanobacterial blooms. Natural transformations are frequently observed in most cyanobacteria of the genera of *Synechococcus* and *Synechocystis*. They naturally absorb DNA from the environment, but are not suitable for the transformation of large DNA [55]. Electroporation can be used for moderately large plasmid genetic operations of cyanobacteria, while the weaknesses include small application scope and low efficiency [93]. *E. coli*-mediated conjugation is a good tool for transferring large plasmids, but the target DNA first needs to be transferred into *E. coli* [43,44,93]. However, we observed that there may be some genes that are toxic to *E. coli* on 351–15,930 bp of the A-4L genome. We blasted the protein sequences of the genes of these A1–A3 regions on the A-4L genome through the NCBI website, but there was no new progress observed in the functional annotation of its genes (Table S9). Taken together, *E. coli*-mediated conjugation may be the most appropriate method to transfer a full-length artificial cyanophage genome into cyanobacteria cells; meanwhile, the issue of DNA cytotoxicity to *E. coli* needs to be addressed, which may be one of the reasons why artificial synthetic cyanophages have not been realized to date. In order to address this issue, the specific toxic genes on the A-4L genome should be identified and then controlled by inducible promoters in the future.

In general, the early and middle gene products encoded by phages usually inhibit or redirect host target proteins, or assist in late gene transcriptions [94]. Unlike marine cyanopodovirus, the genome of freshwater cyanophage A-4L lacks gene-encoding RNA polymerase, and there are many putative bacterial promoter sequences preceding the early and late gene transcriptions, indicating that the expressions of these genes may be host-dependent on RNA polymerase [40]. In this study, given that Syn-A-4-8 only contained structural protein genes and unknown functional genes on the right arm of the A-4L genome, but lacked host-takeover and DNA replication genes, no significant effects could be observed under normal conditions for LS-02. In addition, the promoters in Syn-A-4-8, which were designed and assembled based on the A-4L genome, may not be fully recognized in non-host cyanobacterial Syn7942, as only several transcripts could be observed via transcriptome. Thus, in addition to the attempt to introduce a full-length artificial cyanophage genome into Syn7942, the promoters for the specific ORFs on Syn-A-4-8 could be replaced by common promoters used in Syn7942 in the future.

Although no phenotypes could be observed for LS-02 under normal conditions, its tolerance to salt was found to be altered in this study. The exposure of living cells to high salinity conditions produced two stress reactions, i.e., increasing osmotic pressure and increasing inorganic ion concentrations. Under salt stress conditions, cyanobacterial cells regulate osmotic pressure by accumulating compatible solutes as osmotic protectants [85,95], exclude excess Na^+^ through the Na^+^/H^+^ antiporter and actively absorb K^+^ through the K^+^ transport system [80]. According to the results of the transcriptomic analysis, it is speculated that the heterologous expression of Syn-A-4-8 reduces the ability of LS-02 cells to exclude Na^+^, as several Na^+^/H^+^ antiporter encoding genes are down-regulated in LS-02 compared to those in LS-01, which is not conducive to the process of cell metabolism. In addition, cellular processes, such as sulfur metabolism, ion transport, gene transcription and translation, were also adversely affected, resulting in a prolonged lag phase of LS-02 compared to LS-01 under salt stress conditions.

The growth curves of Syn7942 strains (LS-03, LS-4, LS-5 and LS-6) and the transcriptional levels of cyanophage genes in the Syn7942 strains indicated that the growth differences may be related to the co-effect of *ORF25–26* expression. In addition, *ORF36* and *ORF37* had high transcriptional levels under normal conditions, but the transcriptional levels significantly reduced under 290-millimolar NaCl stress. Therefore, it is speculated that the reduced expressions of *ORF36–37* combined with high expressions of *ORF25–26* may be one of the reasons for the growth lag of LS-02 under 290-millimolar NaCl stress.

## 5. Conclusions

Using *S. cerevisiae* as the chassis, we successfully assembled a truncated cyanophage genome, Syn-A-4-8, based on the natural cyanophage A-4L (41,750 bp) and infected filamentous *Nostoc* sp. PCC 7120. We found potential genes toxic to *E. coli* that exist in the A-4L genome. Syn-A-4-8 was transferred into non-host unicellular cyanobacteria Syn7942 by *E. coli*-mediated conjugation. Then, we investigated the heterologous expression of Syn-A-4-8 in non-host cyanobacteria by using transcriptomic analysis. The growth of the cells of LS-02 exhibited a more prolonged lag phase than that of LS-01 under 290-millimolar NaCl stress. The growth difference caused by the heterologous expression of Syn-A-4-8 in non-host cyanobacterial cells was expounded from the transcription level, and verified that the growth difference may be related to the co-effect of *ORF25* and *ORF26*. This study revealed the expression of a truncated cyanophage genome in heterologous hosts, and lays a foundation for research into the heterologous infection of cyanophages.

## Figures and Tables

**Table 1:** The constructed Syn7942 strains and plasmids used in this study.

Strains/Plasmids	Characteristics ^1,2^
**Plasmids (Size/bp)**	
pJA2 (6537)	Km^R^, *E. coli*-cyanobacteria shuttle vector
Syn-A-4-8 (34,770)	pJA2 harboring the A4-A8 cassette
pSJ03 (20,889)	pJA2 harboring the *ORF25*, *ORF26*, *ORF27* and *ORF38* of the A-4L genome
pSJ04 (14,845)	pJA2 harboring the *ORF25* and *ORF26* of the A-4L genome
pSJ05 (12,253)	pJA2 harboring the *ORF25* of the A-4L genome
pSJ06 (14,243)	pJA2 harboring the *ORF26* of the A-4L genome
**Strains**	
LS-01	Syn7942 harboring the plasmid pJA2, Km^R^
LS-02	Syn7942 harboring genome Syn-A-4-8, Km^R^
LS-03	Syn7942 harboring the plasmid pSJ03, Km^R^
LS-04	Syn7942 harboring the plasmid pSJ04, Km^R^
LS-05	Syn7942 harboring the plasmid pSJ05, Km^R^
LS-06	Syn7942 harboring the plasmid pSJ06, Km^R^

^1^ ORFs include coding and flanging sequences of 800 bp containing original promoters. ^2^ km, kanamycin.

## Data Availability

The data are contained within the article or are available from the authors upon reasonable request.

## Supplementary Materials

The following supporting information can be downloaded at: https://www.mdpi.com/article/10.3390/life12081234/s1, Figure S1: Map of pLS0 plasmid; Figure S2: The gel electrophoresis results of cyanobacteria colony PCR; Figure S3: Full absorption spectrum of strain cells; Figure S4: Pearson correlation coefficient scatter plots; Table S1: Strains and plasmids used in this study; Table S2: Culture conditions for phenotypic test; Table S3: DNA sequences of *CEN6/ARS4* and *His3* markers; Table S4: All the primers used in this study; Table S5: Successful transformation of Syn-A-4-8; Table S6: Sequencing results of Syn-A-4-8; Table S7: The DEGs with functional annotations under normal culture conditions; Table S8: The DEGs with functional annotations under 290-millimolar NaCl stress; Table S9: The result of protein sequences’ alignment within the A1-A3 regions of A-4L genome on the NCBI. References [46,54] are cited in the supplementary materials.
