# Supplementary material for: The Synthesis and Assembly of a Truncated Cyanophage Genome and Its Expression in a Heterogenous Host
Source: Life (Basel). 2022 Aug 15;12(8):1234. doi: 10.3390/life12081234 (PMC9410186; doi:10.3390/life12081234)
Supplement: Supplementary file 1 [file life-12-01234-s001.zip › life-1827983-supplementary.pdf]

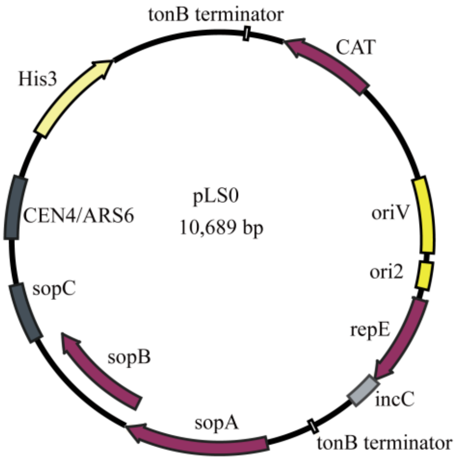

Figure S1. Map of pLS0 plasmid. The chloramphenicol acetyltransferase (CAT).

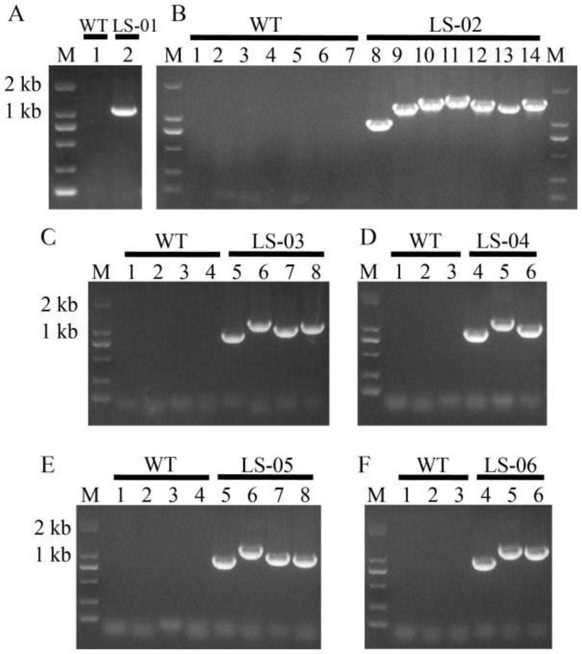

Figure S2. The gel electrophoresis results of cyanobacteria colony PCR. (**A**) WT: lane1, wild type Syn7942. LS-01: lane2, the test group, Syn7942 harboring the plasmid pJA2. The specific primers was F-pJA2/R-pJA2, and the length of PCR products was 1143 bp. (**B**) WT: lane1–7, wild type Syn7942. LS-02: lane8–14, Syn7942 harboring the genome Syn-A-4-8. The specific primers used were from F-T1/R-T1 to F-T7/R-T7. The length of PCR products was 1003 bp, 1351 bp, 1466 bp, 1527 bp, 1446 bp, 1343 bp, 1468 bp respectively. (**C**) WT: lane1–4, wild type Syn7942. LS-03: lane5–8, Syn7942 harboring the plasmid pSJ03. The specific primers used were from F3-T1/R3-T1 to F3-T4/R3-T4. The length of PCR products was 930 bp, 1255 bp, 1098 bp, 1186 bp respectively. (**D**) WT: lane1–3, wild type Syn7942. LS-04: lane4–6, Syn7942 harboring the plasmid pSJ04. The specific primers used were from F4-T1/R4-T1 to F4-T3/R4-T3. The length of PCR products was 963 bp, 1255 bp, 1098 bp respectively. (**E**) WT: lane1–4,

wild type Syn7942. LS-05: lane5–8, Syn7942 harboring the plasmid pSJ05. The specific primers used were from F5-T1/R5-T1 to F5-T4/R5-T4. The length of PCR products was 963 bp, 1255 bp, 1098 bp, 1032 bp respectively. (**F**) WT: lane1–3, wild type Syn7942. LS-06: lane4–6, Syn7942 harboring the plasmid pSJ04. The specific primers used were from F6-T1/R6-T1 to F6-T3/R6-T3. The length of PCR products was 963 bp, 1255 bp, 1211 bp respectively. M: DL2000 DNA Marker.

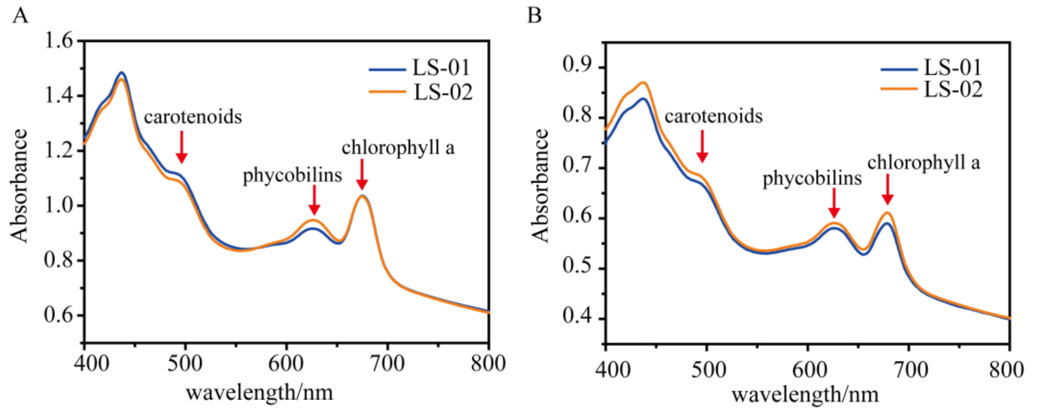

Figure S3. Full absorption spectrum of strain cells. (**A**) Full absorption spectrum of strain cells in BG11 liquid medium without sodium nitrate (BG-N⁻). (**B**) Full absorption spectrum of strain cells in BG11 liquid medium supplemented with salt (290 mM NaCl).

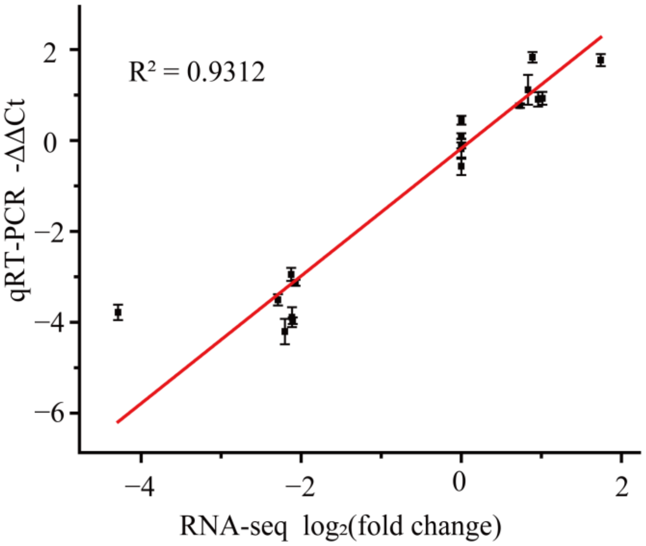

Figure S4. Pearson correlation coefficient scatter plots.

Table S1. Strains and plasmids used in this study.

Name	Characteristics [a]	References
**strains**		
NEB 10-beta Electrocompetent cells	*araD139Δ(ara,leu)7697 fhuA lacX74 galK16 galE15 mcrA80d(lacZM15)recA1 relA1 endA1 nupG rpsL rph spoT1Δ(mrrhsdRMS-mcrBC)*	Biomed, China
HB101	*supE44, Δ(mcrC-mrr), recA13, ara−14, proA2, lacY1, galK2, rpsL20, xyl−5, mtl−1, leuB6, thi-1*	Takara Bio
LE-B01	*E. coli* Top10: pUC57 harboring the B1 cassette	This study
LE-B02	*E. coli* Top10: Pjet1.2 harboring the B2 cassette	This study
LE-B03	*E. coli* Top10: pUC57 harboring the B3 cassette	This study
LE-B04	*E. coli* Top10: pUC57 harboring the B4 cassette	This study
LE-B05	*E. coli* Top10: pUC57 harboring the B5 cassette	This study
LE-B06	*E. coli* Top10: pUC57 harboring the B6 cassette	This study
LE-B07	*E. coli* Top10: pUC57 harboring the B7 cassette	This study
LE-B08	*E. coli* Top10: Pjet1.2 harboring the B8 cassette	This study
LE-B09	*E. coli* Top10: pUC57 harboring the B9 cassette	This study
LE-B10	*E. coli* DH10B:pUC57 harboring the B10 cassette	This study
LE-A04	*E. coli* Top10: pUC57 harboring the A4 cassette	This study
LE-A05	*E. coli* Top10: pUC57 harboring the A5 cassette	This study
LE-A06	*E. coli* Top10: pUC57 harboring the A6 cassette	This study
LE-A07	*E. coli* Top10: pUC57 harboring the A7 cassette	This study
LE-A08	*E. coli* Top10: pUC57 harboring the A8 cassette	This study
LE-01	*E. coli* DH10B harboring genome Syn-A-4-8	This study
BY4741	*MATa his3Δ1 leu2Δ0 met15Δ0 ura3Δ0*	This laboratory
LB-01	BY4741 harboring genome Syn-A-4-8	This study
Syn7942	wild type *Synechococcus elongatus* PCC 7942	This laboratory
LS-01	Syn7942 harboring the plasmid pJA2	This study
LS-02	Syn7942 harboring genome Syn-A-4-8	This study
LS-03	Syn7942 harboring the plasmid pSJ03	This study
LS-04	Syn7942 harboring the plasmid pSJ04	This study
LS-05	Syn7942 harboring the plasmid pSJ05	This study
LS-06	Syn7942 harboring the plasmid pSJ06	This study
**Plasmids (Size/bp)**		
pRS415 (6021)	Leu selection marker, Ap^R, *E. coli*-yeast shuttle vector	[1]
pLS0 (10689)	His3 selection marker, Cm^R, *E. coli*-yeast shuttle vector	This laboratory
pJA2 (6537)	Km^R, *E. coli*-cyanobacteria shuttle vector	[2]
Syn-A1 (11,678)	pRS415 harboring the A1 cassette	This study
Syn-A2 (11,498)	pRS415 harboring the A2 cassette	This study
Syn-A3 (11,298)	pRS415 harboring the A3 cassette	This study
Syn-A-4-8 (34,770)	pJA2 harboring the A4-A8 cassette	This study
pSJ03 (20,889)	pJA2 harboring the *ORF25*, *ORF26*, *ORF27* and	This study

	ORF38 of A-4L genome	
pSJ04 (14,845)	pJA2 harboring the *ORF25* and *ORF26* of A-4L genome	This study
pSJ05 (12,253)	pJA2 harboring the *ORF25* of A-4L genome	This study
pSJ06 (14,243)	pJA2 harboring the *ORF26* of A-4L genome	This study

[a] *Ap*, ampicillin; *Cm*, chloramphenicol; *Km*, kanamycin.

Table S2. Culture conditions for phenotypic test.

**liquid medium**	**light density (μmol/(m²·s))**	**Temperature (°C)**
BG11	200	37
BG11	100	37
BG11	40	37
BG11	40	30
BG-N⁻ [a]	100	37
BG11 medium supplemented with 190 mM NaCl	100	37
BG11 medium supplemented with 290 mM NaCl	100	37

[a] BG-N⁻: BG-11 medium without sodium nitrate

Table S3. DNA sequences of *CEN6/ARS4* and *His3* marker.

**Primers**	**Sequences (5'-3')**
CEN6/ARS4	gatcgcttgcctgtaacttacacgcgcctcgtatcttttaatgatggaataatttgggaatttactct<br>gtgtttatttatttttatgttttgtatttggattttagaaagtaaataaagaaggtagaagagttacg<br>gaatgaagaaaaaaaaataaacaaaggtttaaaaaatttcaacaaaaagcgtactttacatata<br>tatttattagacaagaaaagcagattaaatagatatacattcgattaacgataagtaaaatgtaa<br>aatcacaggattttcgtgtgtggtcttctacacagacaagatgaaacaattcggcattaatacct<br>gagagcaggaagagcaagataaaaggtagtatttgttggcgatcccctagagtcttttacatc<br>ttcggaaaacaaaaactattttttctttaatttctttttttactttctatttttaatttatatatttatattaa<br>aaaatttaaattataattatttttatagcacgtgat
***His3* marker** [a]	ctagtacactctatatttttttatgcctcggtaatgattttcattttttttttttccacctagcggatgact<br>cttttttttttcttagcgattggcattatcacataatgaattatacattatataaagtaatgtgatttctt<br>cgaagaatatactaaaaaatgagcaggcaagataaacgaaggcaaagatgacagagcaga<br>aagccctagtaaagcgtattacaaatgaaaccaagattcagattgcgatctctttaaagggtgg<br>tcccctagcgatagagcactcgatcttcccagaaaaagaggcagaagcagtagcagaacagg<br>ccacacaatcgcaagtgattaacgtccacacaggtatagggtttctggaccatatgatacatgct<br>ctggccaagcattccggctggtcgctaatcgttgagtgcattggtgacttacacatagacgacc<br>atcacaccactgaagactgcgggattgctctcggtcaagcttttaaagaggccctactggcgcg<br>tggagtaaaaaggtttggatcaggatttgcgcctttggatgaggcactttccagagcggtggta<br>gatctttcgaacaggccgtacgcagttgtcgaacttggtttgcaaagggagaaagtaggagat<br>ctctcttgcgagatgatcccgcattttcttgaaagctttgcagaggctagcagaattaccctccac<br>gttgattgtctgcgaggcaagaatgatcatcaccgtagtgagagtgcgttcaaggctcttgcgg

	ttgccataagagaagccacctcgcccaatggtaccaacgatgttccctccaccaaaggtgttctt atgtag

[a] The *His3* Marker DNA sequence contains the His3 promoter sequence.

Table S4. All the primers used in this study.

Primers	Sequences (5'-3')
**Primers involved in the validation for Syn-A-4-8**	
F-T1	GCCAGTTTAGTCTGACCATCTC
R-T1	CAGCACTACCGTCTAACACTC
F-T2	ATATGTCAGCAGTTCTACGAGACTGGC
R-T2	ACACACAAAACACATGGTTAGTGATGT
F-T3	ACCCTGTTGCAGTAGGTTATCTGTTG
R-T3	CACACAACGTTAAACAAGGTCAACGT
F-T4	TCTGTCACCACTGTAGTCATAGCCTC
R-T4	TATACTCACACGTCAGGCTGTGTTCC
F-T5	AATGTGTGTGTGTTAGTGTAGGTGGTC
R-T5	AACACTGGGACAGGAAACCTTATCAG
F-T6	AGTGTTCAGTGGTGAGTATGTGAATGT
R-T6	GGGCCTCGTGATACGCCTATTTTTATAG
F-T7	CTAGCAGAATTACCCTCCACGTTGATTG
R-T7	TAGCCTACGAGACAGCACATTAACAATG
**Primers involved in the validation for pJA2**	
F-pJA2	CCAGTGTTACAACCAATTAACCAATTCTGA
R-pJA2	CAATTGATTACCGCCTTTGAGTGAGC
**Primers involved in the validation for pSJ03**	
F3-T1	ACTACGGTTATTAACCATCTTCCGT
R3-T1	ACAACCGAAGATAAAGACACCTG
F3-T2	ATGATCTCCGAGCGGATCAAT
R3-T2	GCTTGTCCTTGCGGTTGGT
F3-T3	ATTAGGCTATGACTGGGCACAACAGA
R3-T3	CTGCTGCTTTAACTGGTGGT
F3-T4	CGTAGTTGTACCGTTCCAGGT
R3-T4	CCGTTCAGTCTGAGAGTAAAGCT
**Primers involved in the validation for pSJ04**	
F4-T1	CCAATGGCATCTGTCTCATCTAG
R4-T1	GATAACCGTCCATTGAACGGAAAT
F4-T2	ATGATCTCCGAGCGGATCAAT
R4-T2	GCTTGTCCTTGCGGTTGGT
F4-T3	ATTAGGCTATGACTGGGCACAACAGA
R4-T3	CTGCTGCTTTAACTGGTGGT
**Primers involved in the validation for pSJ05**	
F5-T1	CCAATGGCATCTGTCTCATCTAG
R5-T1	GATAACCGTCCATTGAACGGAAAT

F5-T2	ATGATCTCCGAGCGGATCAAT
R5-T2	GCTTGTCCTTGCGGTTGGT
F5-T3	ATTAGGCTATGACTGGGCACAACAGA
R5-T3	CTGCTGCTTTAACTGGTGGT
F5-T4	CCAGGGGTTTGGTTATAACCAGGAT
R5-T4	CGTCAGCAACGTACAGCTATTACT
**Primers involved in the validation for pSJ06**	
F6-T1	CCAATGGCATCTGTCTCATCTAG
R6-T1	GATAACCGTCCATTGAACGGAAAT
F6-T2	ATGATCTCCGAGCGGATCAAT
R6-T2	GCTTGTCCTTGCGGTTGGT
F6-T3	GCTCTAGGGTCACCTGTGAGT
R6-T3	CGTTGTTAGGAATGTACACACCT
**Primers for qRT-PCR**	
F-rnpB	CCACAGAAACATACCGCCGA
R-rnpB	GGTTTACCGAGCCAACACCT
F-25orf	ACACGAAACACCAACGCATC
R-25orf	TTGCTGCTCTTTCTGGTGCT
F-26orf	TCTCGGCTATCTCGTCCGAT
R-26orf	AAGTCAAGGCTCTCAACCCG
F-27orf	GGCTGCATACCTTCTGGGTT
R-27orf	CCATCAGCACCTCGTCAACT
F-36orf	CGTCTCACGTTTTGCAGGTG
R-36orf	GCCCAGGCTATTCAGGTTGT
F-37orf	AGTACCTGCGGATGCTGATG
R-37orf	TCACGATGACTGATGCCGAG
F-38orf	AGCCTCTCGTTCGTTCTGTG
R-38orf	ACGAACCTACCACTGAGGAAAT
F-0274	AGTCCTCAGCATCAAGAACGC
R-0274	CAGGCTGAAAAGCAACTAAGAGA
F-0303	GTTTGTGGCTTTGGTCGCAT
R-0303	GGATGGCTTCGTCTGTAGCA
F-1205	ATGTTCCGCCAGTCCTGC
R-1205	ACGCACCGTGTTTTGAATGT
F-1952	TATCGCAAGACTGGTGGCAT
R-1952	TTGCACTTCTGTCAGCGAGA
F-2462	TGAGGGCATTGTGATAGTCGC
R-2462	TTGCGGATTTAGCAGATTGGGT
F-1457	TTTACGGGGCGGGTAATGTC
R-1457	TAGGCACCGTAGAGACGGAT
F-1152	GGGCCATACCAAGAAGCCAT
R-1152	ATCGTCACCAATCGTCTCCG
F-0581	GGTCGTTGACCAGGACTGAG
R-0581	TTGACGCTCCAACTGAGCAA

F-0608	GCTTGCTCAACAAGGACTGC
R-0608	AACGGTGATGTAACCGCCTT
F-0620	TGACCACCACGGGAATTACG
R-0620	CGATACGAGTGCTCCCAGAC
F-1682	CCCTCTCCTTCACTTGGCTG
R-1682	GGAAGCCGACTTGAGGAACA
F-1683	TCAGCCGTTGATGGGAGATG
R-1683	GTCAAACCAGCCGTCTTCCT
F-1685	CAGTACCCTTGGGGCTAACG
R-1685	CGGTGATACTGCCAGAGACC
F-1686	TGTTGTCGATAAGCGCGGTA
R-1686	GCGTTGGAAAACTTGGCGAT
F-1689	CTTGCAGTACCTCGTGCTCA
R-1689	ACCAGTTTCAACCGGCAGAT
F-1690	TGGCCTTTCTTGCCTCTTCC
R-1690	CAAGCTCAGTACCCCCAAGC
F-1978	CATTGTTCGAGGCAGGGAGT
R-1978	GTCACAGCGCAAACCTTCAG
F-2580	GCGCTGCTGTCTCTTGATTG
R-2580	TTTCCCTCGACTTTGGCCTC

Table S5. Successful transformation of Syn-A-4-8.

Plasmid (characteristics [a])	LB solid medium	Transformants (number [d])
pUC19 (Ap^R) [b]	LB + Ap	97 (100 times dilution)
H_2O	LB + Ap	- (negative control)
H_2O	LB + Km	- (negative control)
Syn-A1 (Ap^R) [c]	LB + Ap	-
Syn-A2 (Ap^R) [c]	LB + Ap	-
Syn-A3 (Ap^R) [c]	LB + Ap	-
Syn-A-4-8 (Km^R) [c]	LB + Km	39

[a] Ap, ampicillin; Km, kanamycin. [b] pUC19, positive control, 0.5 ng of DNA. [c] Plasmids were extracted from 5 mL of yeast colonies and used to transform *E. coli*. [d] -, no transformant.

Table S6. Sequencing results of Syn-A-4-8.

**Locations**	**Base mutation**	**Codon mutation**
3806	G->T	Ser->Ile
9424	C->A	Pro->Gln
21,058	A->G	Asn->Ser
21,107	C->A	Thr->Lys
23,334	G->A	Gly->Arg

Table S7. The DEGs with functional annotations under normal culture conditions.

GeneName	$log_2(FC)$ [b]	*p*-value	product
**Group 1: DEGs involved in oxidative phosphorylation**			
Syn7942_2601 [a] (*ctaA*)	-0.9313	0.0060	putative cytochrome aa3 controlling protein, CtaA, HAS
Syn7942_2603 (*coxA*)	-0.5876	0.0473	Cytochrome-c oxidase, CO Ⅰ
Syn7942_2604 (*coxC*)	-0.9105	0.0055	Cytochrome-c oxidase subunit III, COⅢ
**Group 2: DEGs involved in two-component system**			
Syn7942_2601 [a] (*cox15*)	-0.9313	0.0060	putative cytochrome aa3 controlling protein
Syn7942_0857	-0.6596	0.0336	CheW protein
Syn7942_0855	-0.6327	0.0464	response regulator receiver domain protein (CheY-like)
**Group 3: DEGs involved in heat shock proteins**			
Syn7942_2401	-0.7721	0.0133	heat shock protein HSP20, IpbA
Syn7942_2468 (*dnaK*)	-1.3031	0.0165	Heat shock protein HSP70, DnaK
Syn7942_1813	-0.6975	0.0284	Heat shock protein HSP90, HtpG
Syn7942_0685	-0.7910	0.0283	Chaperonin Cpn60/TCP-1, HSP60
Syn7942_1809	-1.2614	0.0038	ATP-binding subunits of Clp protease, ClpA
**Group 4: DEGs involved in photosynthesis**			
Syn7942_0407	0.6889	0.0266	photosystem I reaction center subunit X, PsaK
Syn7942_1479 (*petG*)	0.7279	0.0410	cytochrome b6-f complex subunit 5, PetG
**Group 5: DEGs encoding in transporters**			
Syn7942_1472	-0.8543	5.60×10^{-5}	conserved hypothetical protein (MnhE, Multisubunit Na^+/H^+ antiporter, MnhE subunit)
Syn7942_1473	-0.8596	1.21×10^{-5}	putative multicomponent $Na^+:H^+$ antiporter subunit D

[a] These DEGs belong to both group 1 and group 2. [b] log_2 (FC), log_2 (Fold Change).

Table S8. The DEGs with functional annotations under 290 mM NaCl stress.

GeneName	$log_2(FC)$ [b]	*p*-value	product
**Group 1: DEGs involved in sulfur metabolism**			
Syn7942_0019	-0.7031	0.0001	sulfite reductase (ferredoxin)
Syn7942_1689	-2.1059	2.19×10^{-25}	Rhodanese-like
Syn7942_1680 [a] (*cysA*)	-1.7312	1.37×10^{-20}	Sulphate transport system permease protein 1
Syn7942_1681 [a] (*cysP*)	-2.7096	6.40×10^{-35}	Thiosulphate-binding protein
Syn7942_1682 [a]	-2.1142	5.81×10^{-21}	Sulphate transport system permease protein 2
Syn7942_1685 [a] (*cysW*)	-2.1253	1.25×10^{-41}	Sulphate transport system permease protein 2
Syn7942_1686 [a] (*cysP*)	-2.0713	9.35×10^{-37}	Thiosulphate-binding protein
Syn7942_1687 [a] (*cysT*)	-1.8290	2.39×10^{-25}	Sulfate ABC transporter, permease protein CysT
Syn7942_1688 [a] (*cysW*)	-1.9636	5.44×10^{-36}	Sulfate ABC transporter, permease protein CysW
Syn7942_1722 [a] (*cysP*)	-0.9125	9.75×10^{-13}	Thiosulphate-binding protein
**Group 2: DEGs involved in carbohydrate metabolism**			
Syn7942_0603	0.6646	0.0001	glucose-1-phosphate adenylyltransferase, GlgC
Syn7942_0781	0.6419	0.0001	phosphoenolpyruvate synthase
Syn7942_0808	0.6148	5.71×10^{-8}	Sucrose phosphate phosphatase, SPP
Syn7942_2425	0.8701	0.0001	chaperon-like protein for quinone binding in photosystem II
**Group 3: DEGs involved in photosynthesis antenna proteins and photosynthesis**			
Syn7942_0240	0.8101	5.65761×10^{-6}	allophycocyanin-B
Syn7942_0328	0.5911	4.75392×10^{-5}	phycobilisome core-membrane linker polypeptide
Syn7942_1048	0.6175	0.0031	Phycocyanin, alpha subunit
Syn7942_0327	0.5952	0.0289	allophycocyanin alpha chain
Syn7942_0893	1.4858	5.21081×10^{-11}	photosystem q(b) protein
Syn7942_1389	0.8526	9.7026×10^{-8}	photosystem q(b) protein
Syn7942_2010	0.9648	1.83799×10^{-7}	cytochrome c550
Syn7942_0697	0.7251	7.39184×10^{-7}	photosystem II core light harvesting protein

Syn7942_0239	0.9733	8.839×10^{-7}	cytochrome C6 soluble cytochrome f
Syn7942_2331	0.6479	0.0001	cytochrome b6
Syn7942_2332	0.6154	0.0002	cytochrome b6-f complex subunit 4
Syn7942_0696	0.6060	0.0022	photosystem II PsbT protein
Syn7942_1637	0.6924	0.0090	photosystem II D2 protein (photosystem q(a) protein)
**Group 4: DEGs encoding in transporters**			
Syn7942_1469	-0.6996	2.40×10^{-5}	putative multicomponent Na+:H+ antiporter subunit B
Syn7942_1472	-0.8543	5.60×10^{-5}	conserved hypothetical protein (MnhE, Multisubunit Na+/H+ antiporter, MnhE subunit)
Syn7942_1473	-0.8596	1.21×10^{-5}	putative multicomponent Na+:H+ antiporter subunit D
Syn7942_1474	-0.7922	0.0014	putative multicomponent Na+:H+ antiporter subunit C
Syn7942_2175 (*fbpA*)	-0.9407	5.83×10^{-6}	transport system substrate-binding protein
Syn7942_2492 (l*ivF*)	-0.8056	1.26×10^{-5}	ATPase
Syn7942_2493 (*livG*)	-0.7270	1.40×10^{-5}	ATPase
Syn7942_2105 (*nrtC*)	-0.6895	0.0001	nitrate transport ATP-binding subunits C and D
**Group 5: DEGs encoding in heat shock protein and signal transduction mechanisms**			
Syn7942_2306	-0.9547	4.14×10^{-6}	DnaJ-like heat shock protein
Syn7942_1684	-1.9449	1.11098×10^{-23}	cAMP receptor protein (CRP)
Syn7942_1158	-0.6212	1.0819×10^{-6}	c-di-GMP phosphodiesterase class I (EAL domain)
**Group 6: DEGs involved in oxidative phosphorylation**			
Syn7942_1766	-0.7359	1.33×10^{-10}	cytochrome bd ubiquinol oxidase subunit II, CydB
Syn7942_1344	-0.5860	0.0002	NAD(P)H-quinone oxidoreductase subunit I, NdhI
Syn7942_0198	-0.7544	3.27×10^{-12}	Type II NADH dehydrogenase (NDH-2)
**Group 7: DEGs involved in transcription and translation**			
Syn7942_1111	-1.5237	1.3707×10^{-15}	serine/threonine protein kinase
Syn7942_1310	-0.7195	2.66044×10^{-6}	ndhF3 operon transcriptional regulator
Syn7942_1710 (*rpoZ*)	-0.7148	2.5498×10^{-7}	RNA polymerase subunit omega
Syn7942_1739	-0.6917	0.0011	transcriptional regulator, MerR family

Syn7942_1534	-1.0316	7.04691×10^{-25}	conserved hypothetical protein
Syn7942_0065	-0.6103	8.00377×10^{-5}	bacterial translation initiation factor 3 (bIF-3)
Syn7942_2223	-0.6534	2.51957×10^{-9}	SSU ribosomal protein S17P
Syn7942_1112	-0.7082	0.0012	ribosomal large subunit pseudouridine synthase D
Syn7942_2203	-0.8445	4.36122×10^{-11}	bacterial peptide chain release factor 1 (bRF-1)

[a] These DEGs belong to both group 1 and group 2. [b] $\log_2$ (FC), $\log_2$ (Fold Change).

Table S9. The result of protein sequences' alignment within the A1-A3 regions of A-4L genome on the NCBI.

ORF	Predicted protein [a]	Accession no. [a]	Homologues in cyanophage	Accession no. [b]	Percent Identity	*E*-value	Predicted protein [b]
ORF1	hypothetical protein	YP_009042771.1	-	-	-	-	-
ORF2	hypothetical protein	YP_009042772.1	-	-	-	-	-
ORF3	hypothetical protein	YP_009042773.1	S-CBP42	YP_009220220.1	31.90%	3e-18	hypothetical protein AVU76_gp36
ORF4	hypothetical protein	YP_009042774.1	-	-	-	-	-
ORF5	hypothetical protein	YP_009042775.1	-	-	-	-	-
ORF6	hypothetical protein	YP_009042776.1	-	-	-	-	-
ORF7	hypothetical protein	YP_009042777.1	-	-	-	-	-
ORF8	hypothetical protein	YP_009042778.1	-	-	-	-	-
ORF9	hypothetical protein	YP_009042779.1	-	-	-	-	-
ORF10	hypothetical protein	YP_009042780.1	-	-	-	-	-
ORF11	hypothetical protein	YP_009042781.1	-	-	-	-	-
ORF12	hypothetical protein	YP_009042782.1	-	-	-	-	-
ORF13	hypothetical protein	YP_009042783.1	-	-	-	-	-
ORF14	hypothetical protein	YP_009042784.1	PaV-LD	YP_004957337.1	53.78%	7e-30	KilA-like protein
ORF15	hypothetical protein	YP_009042785.1	-	-	-	-	-
ORF16	DNA primase/helicase	YP_009042786.1	Pf-WMP3	YP_001285774.1	24.74%	1e-23	DNA primase/helicase
ORF17	Endonuclease	YP_009042787.1	S-CBP3	YP_009103517.1	44.83%	4e-27	Endonuclease
ORF18	hypothetical protein	YP_009042788.1	-	-	-	-	-
ORF19	DNA polymerase	YP_009042789.1	PP	YP_008766972.1	28.79%	5e-59	DNA polymerase
ORF20	hypothetical protein	YP_009042790.1	Pf-WMP3	YP_001285782.1	51.19%	6e-15	PfWMP3_17
ORF21	recombination protein	YP_009042791.1	Pf-WMP3	YP_001285785.1	39.10%	1e-25	putative phage associated recombination protein
ORF22	hypothetical protein	YP_009042792.1	-	-	-	-	-
ORF23	hypothetical protein	YP_009042793.1	Pf-WMP3	YP_001285788.1	26.32%	3e-15	PfWMP3_23

[a] The gene related information of A-4L. [b] The gene related information of the homologous cyanophage.

Supplementary References

1. Simons, R.W.; Houman, F.; Kleckner, N. Improved single and multicopy lac-based cloning vectors for protein and operon fusions. *Gene* **1987**, *53*, 85–96, https://doi.org/10.1016/0378-1119(87)90095-3.
2. Anfelt, J.; Hallström, B.; Nielsen, J.; Uhlén, M.; Hudson, E.P. Using transcriptomics to improve butanol tolerance of *Synechocystis* sp. Strain PCC 6803. *Appl. Environ. Microbiol.* **2013**, *79*, 7419–7427, https://doi.org/10.1128/AEM.02694-13.
