# Supplementary figures and images for: The Synthesis and Assembly of a Truncated Cyanophage Genome and Its Expression in a Heterogenous Host
Source: Life (Basel). 2022 Aug 15;12(8):1234. doi: 10.3390/life12081234 (PMC9410186; doi:10.3390/life12081234)

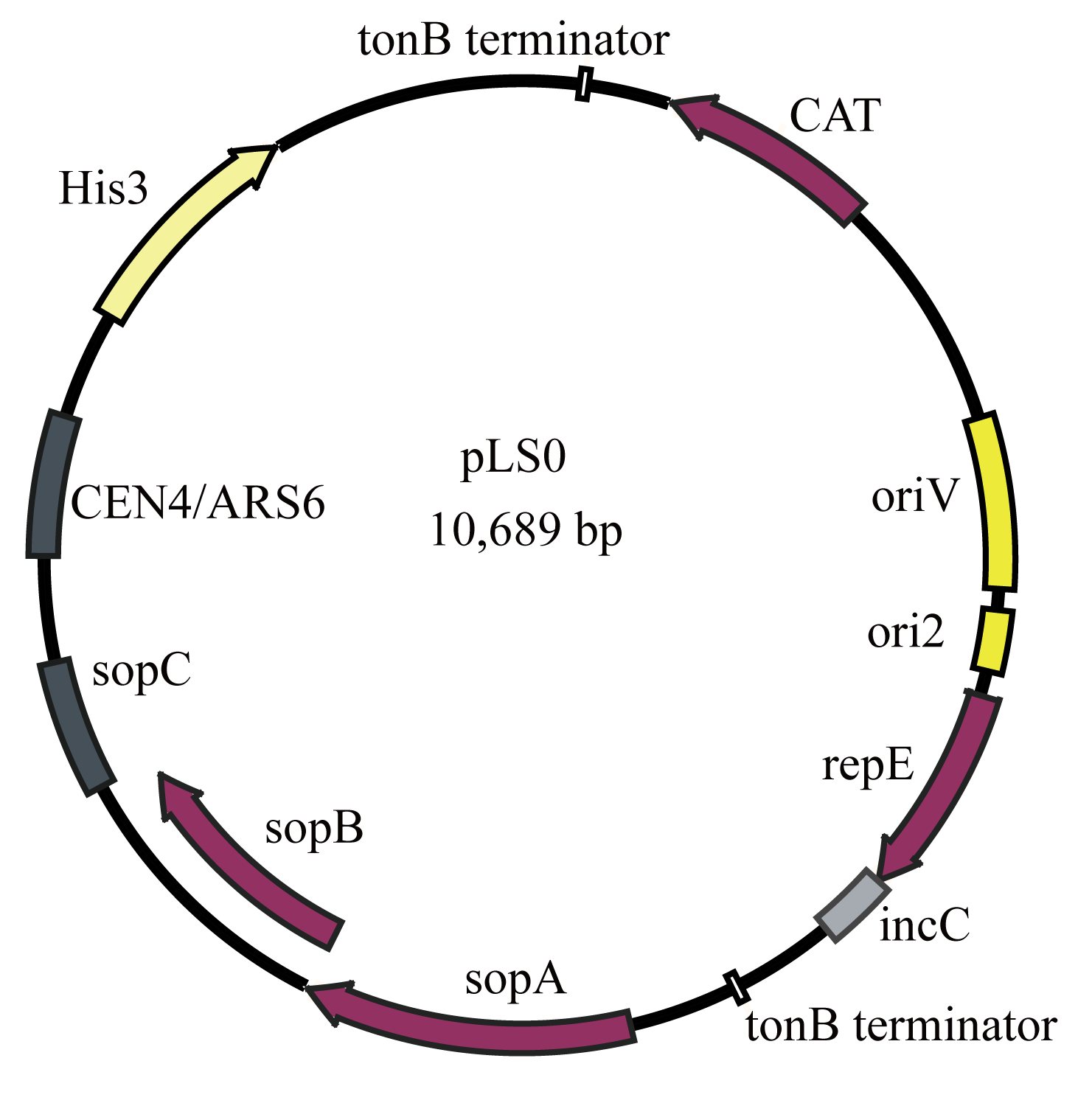

Supplement: Supplementary file 1 [file life-12-01234-s001.zip › Figure S1.tif]

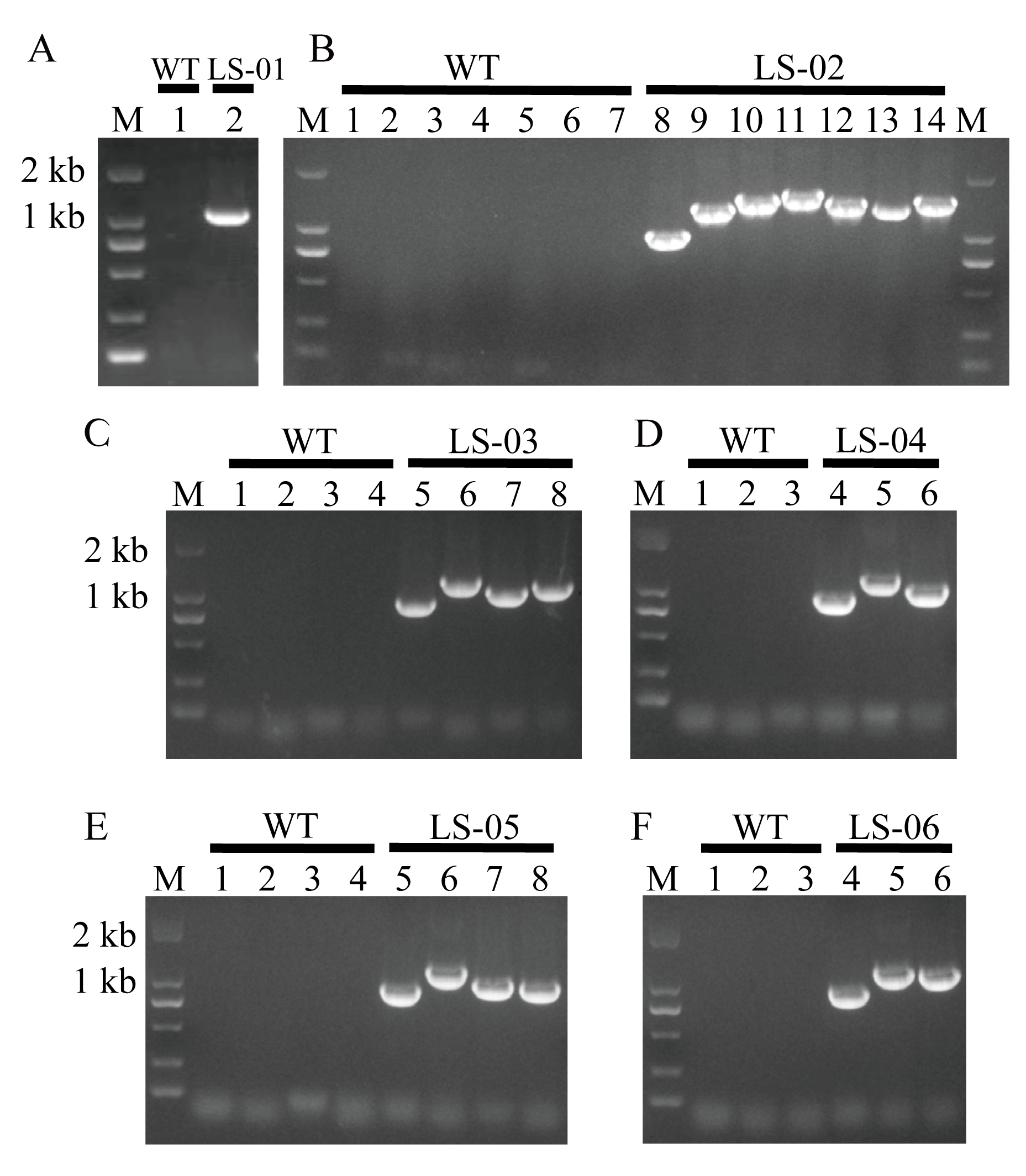

Supplement: Supplementary file 1 [file life-12-01234-s001.zip › Figure S2.tif]

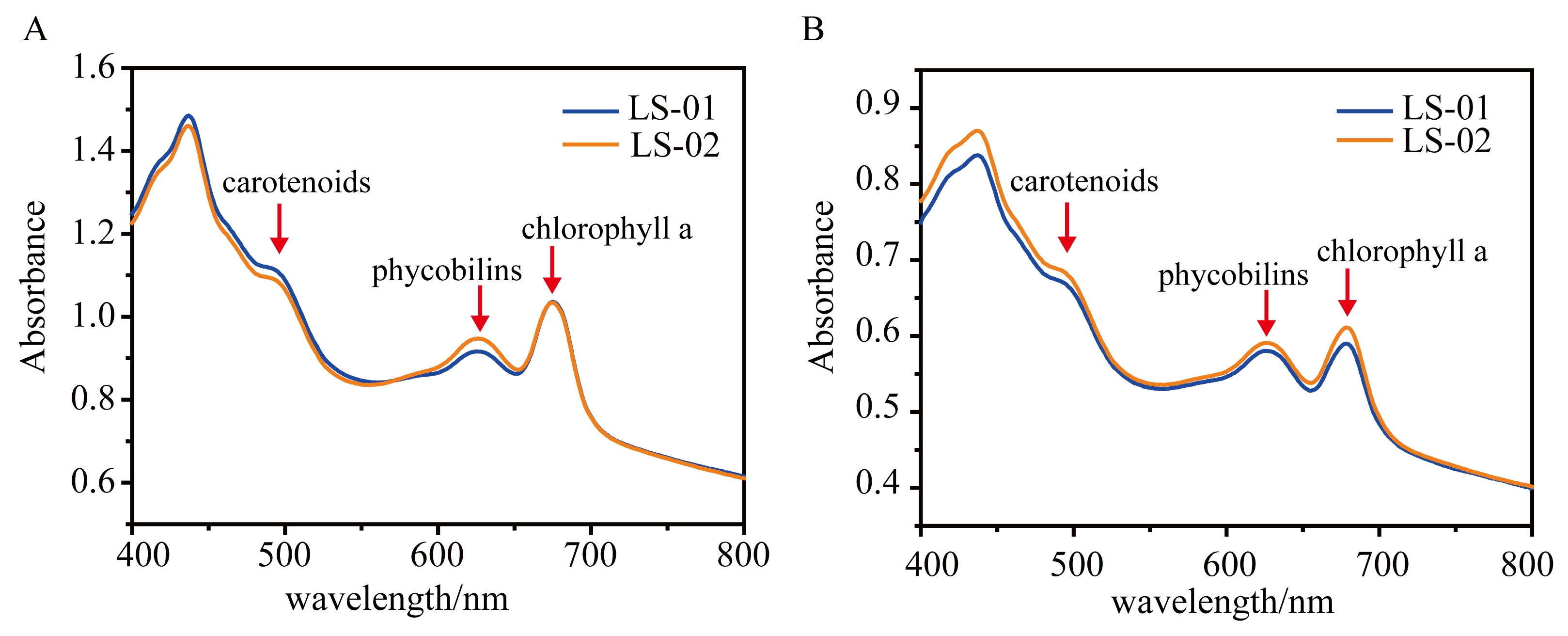

Supplement: Supplementary file 1 [file life-12-01234-s001.zip › Figure S3.tif]

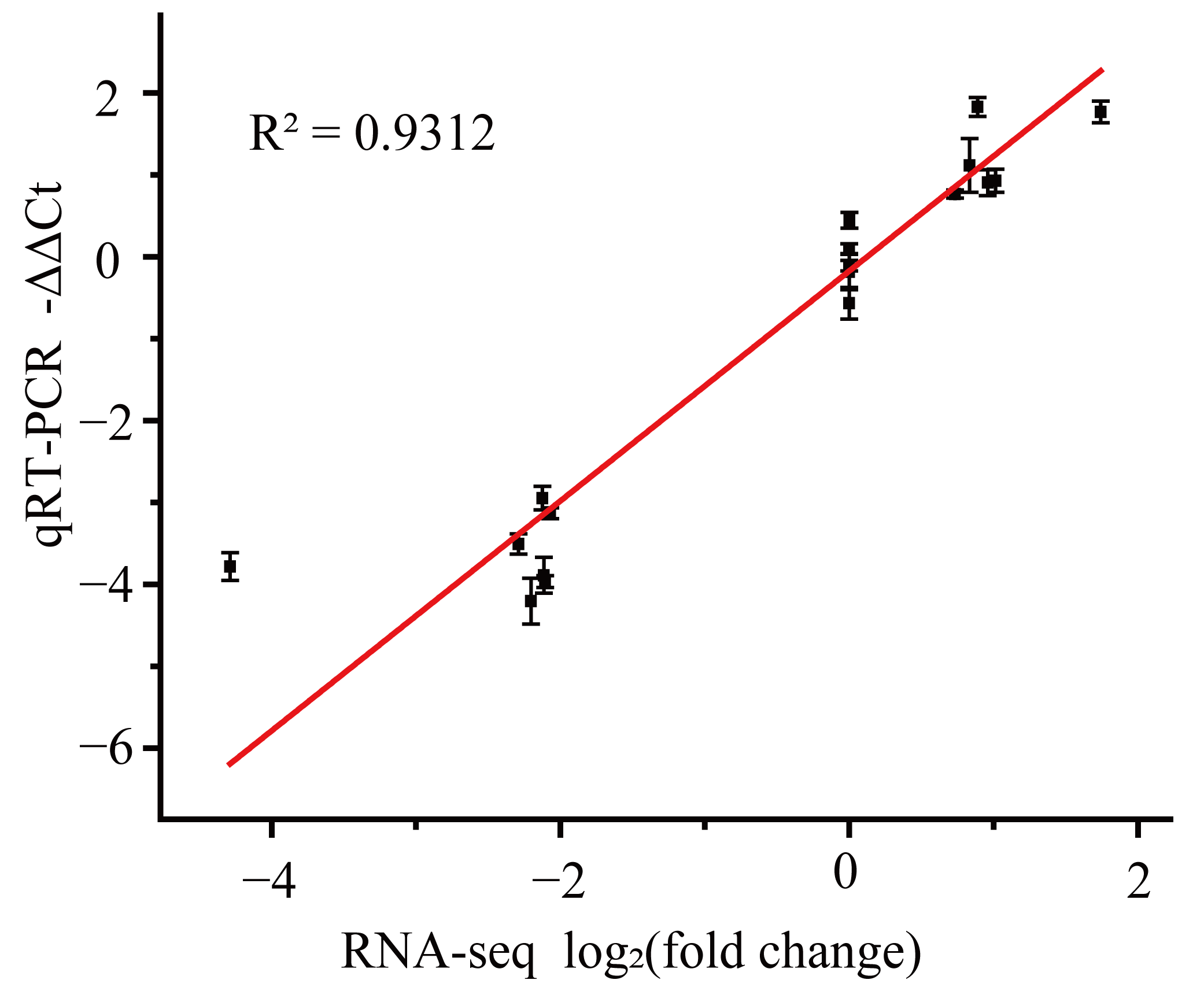

Supplement: Supplementary file 1 [file life-12-01234-s001.zip › Figure S4.tif]
